# Multidimensional protein fractionation using ProteomeLab PF 2D™ for profiling amyotrophic lateral sclerosis immunity: A preliminary report

**DOI:** 10.1186/1477-5956-6-26

**Published:** 2008-09-12

**Authors:** Joshua D Schlautman, Wojciech Rozek, Robert Stetler, R Lee Mosley, Howard E Gendelman, Pawel Ciborowski

**Affiliations:** 1 Department of Pharmacology and Experimental Neuroscience, University of Nebraska Medical Center, Omaha, NE USA; 2 Department of Biochemistry and Molecular Biology, University of Nebraska Medical Center, Omaha, NE USA; 3 Department of Virology, National Veterinary Research Institute, Pulawy, Poland; 4 Beckman Coulter, Inc. 4300 N. Harbor Boulevard, P.O. Box 3100, Fullerton, CA 92834-3100, USA

## Abstract

**Background:**

The ProteomeLab™ PF 2D platform is a relatively new approach to global protein profiling. Herein, it was used for investigation of plasma proteome changes in amyotrophic lateral sclerosis (ALS) patients before and during immunization with glatiramer acetate (GA) in a clinical trial.

**Results:**

The experimental design included immunoaffinity depletion of 12 most abundant proteins from plasma samples with the ProteomeLab™ IgY-12 LC10 column kit as first dimension separation, also referred to as immuno-partitioning. Second and third dimension separations of the enriched proteome were performed on the PF 2D platform utilizing 2D isoelectric focusing and RP-HPLC with the resulting fractions collected for analysis. 1D gel electrophoresis was added as a fourth dimension when sufficient protein was available. Protein identification from collected fractions was performed using nano-LC-MS/MS approach. Analysis of differences in the resulting two-dimensional maps of fractions obtained from the PF 2D and the ability to identify proteins from these fractions allowed sensitivity threshold measurements. Masked proteins in the PF 2D fractions are discussed.

**Conclusion:**

We offer some insight into the strengths and limitations of this emerging proteomic platform.

## Background

Despite technological advances in proteomics, analysis of complex biological samples remains a significant challenge [1]. Some of the most complex biological samples routinely submitted for proteomic profiling include serum or plasma [2-4]. While disease markers abound in plasma are reflective of ongoing disease, complexity of sample due to post-translational modifications (PTMs) and protein isoforms presents major obstacles for biomarker identification [5-8]. Reproducibility, sensitivity, resolution, and high throughput analysis are among the developing areas for proteomic platforms [9]. The need for better separation and analytical techniques cannot be overstated [10].

Improvements in proteomics platforms have been realized in recent years [11,12]. For example, 2D SDS-PAGE when combined with difference in gel electrophoresis (DIGE) was developed as a profiling platform wherein proteins are identified based on electrospray ionization mass spectrometry (ESI-MS/MS) of trypsin-derived peptides. However, resolution of hydrophobic proteins and those within a high molecular mass range is limited [13-15]. This may be overcome using a combination of molecular sieving chromatography with the multi-dimensional protein identification technology (MudPIT) or protein microarrays [16]. Other "bottom-up" tools, such as surface enhanced laser desorption ionization-time of flight (SELDI-TOF) and matrix-assisted labelled desorption/ionization-time of flight (MALDI-TOF), while useful, do not address PTMs in complex body fluids. Another critical need is protein quantification since changes in proteome profiles may be subtle, yet biologically significant [12]. For example, protein glycosylation or phosphorylation leading to functional modification affects only a small percentage of the total protein pool linked to physiological changes. Therefore, fractionating complex protein mixtures while maintaining intact proteins in liquid phase is a most desirable feature for use in further analyses ("top-down proteomics"). Fractions collected in liquid phase would provide simpler and more informative secondary analysis, in contrast to gel-embedded proteins in 2D DIGE [17-19] where intact protein recovery is difficult and associated with greater quantitative loss. Additionally, once protein is enzymatically digested for LC-MS/MS analysis, it cannot be used for other analysis such as Western blot assays.

ProteomeLab™ PF 2D offers an alternative approach to protein profiling that addresses issues of complexity and utilization of fractions after analysis. To assess the utility of the PF 2D platform for proteomic profiling, we compared plasma samples recovered from amyotrophic lateral sclerosis (ALS) patients who were immunized with glatiramer acetate (GA) to those from non-immunized ALS patients. Strengths and weaknesses of this new proteomic platform for biomarker discovery are discussed.

## Results

### Plasma sample immunodepletion

**Figure 1:**
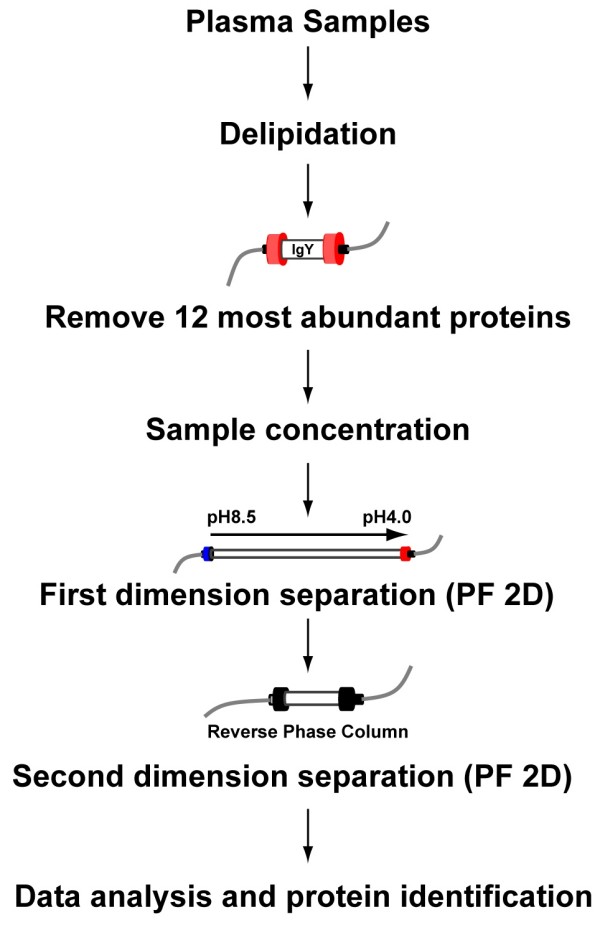
**Experimental design**. Experimental design of multidimensional fractionation using plasma from ALS patients involved in a phase II clinical trial with GA.

**Figure 2:**
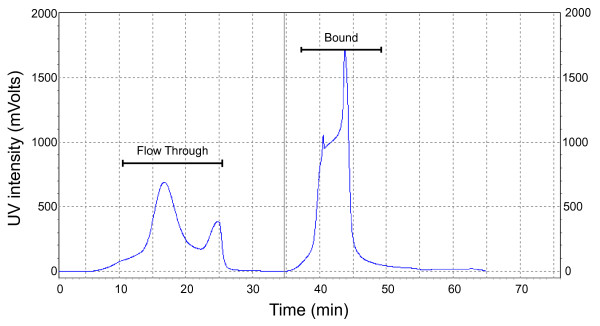
**Immunodepletion**. Chromatography of immunodepletion of plasma using ProteomeLab™ IgY-12. 250 μL of human plasma was partitioned with LC 10 column at an absorbance of 280 nm. The Flow Through was collected (8–27 min) and used for further analysis with PF 2D. The bound fraction was not analyzed.

Patient serum/plasma represents clinical material that is easily obtained with fewer restrictions compared to other sample types, including cerebrospinal fluid or tissue biopsy; thus, it is one of the most commonly tested patient material from which diagnostic tests are performed. Collectively, protein concentrations span a very broad range (10^12^-fold) [20] in serum/plasma from which the differential concentration in individual protein concentrations presents potential targets for the discovery of clinically important biomarkers. However, the presence of very highly abundant proteins and the complexity of plasma proteins present formidable challenges. Twelve of the most abundant proteins comprise ~96% of the total protein mass from human plasma, with albumin comprising approximately 40–50% of protein. Presence of these abundant proteins in plasma samples masks differential levels of low to medium abundant ones. One strategy is to remove the most abundant proteins prior to profiling. To assess that approach, we used immunoaffinity chromatography with a column that is based on IgY technology to selectively remove 12 of the most abundant proteins in human serum/plasma (Figure 1, experimental design). One caveat of immunodepletion is that potential biomarkers that bind to albumin or highly abundant proteins may also be completely or partially depleted from serum samples through protein-protein interactions. However, this possibility can be evaluated with further analyses upon elution of the adherent protein fraction. Although the IgY-12 LC10 affinity column has the highest capacity of commercial immunoaffinity products currently available, only 250 μL of plasma sample can be processed during one chromatographic cycle. Flow through fractions (8–27 minutes) containing unbound proteins (Figure 2) were collected, pooled, concentrated, and submitted for analysis on the PF 2D platform. Protein yields from the flow through fractions were between 0.73 and 3.0 mg per mL of plasma.

### Protein fractionation 2-dimensions (PF 2D)

**Figure 3:**
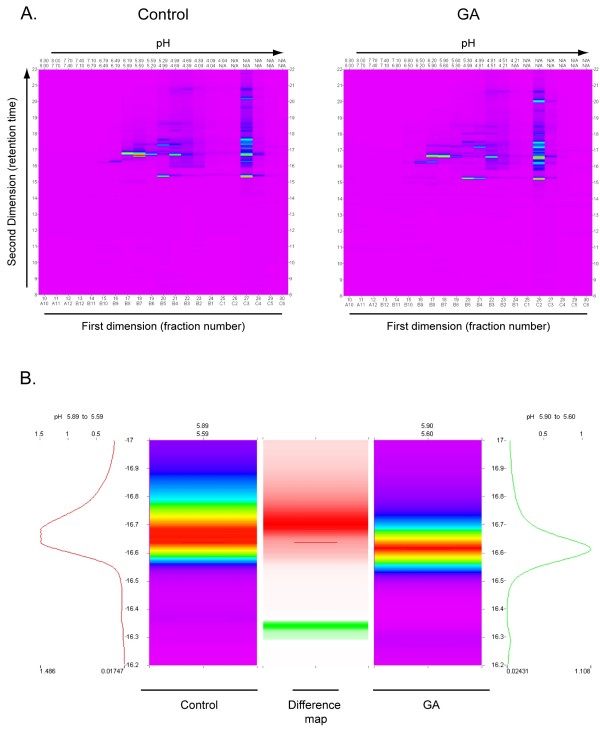
**Results of profiling analysis**. A. PF 2D two-dimensional heat maps of a representative set of samples obtained from one individual before and after GA immunization. PF 2D first dimension separation is based on isoelectric point (pI). PF 2D second dimension separation utilizes reverse phase HPLC fractionation. B. Comparison of two aligned peaks from analyses shown in (A) displaying a quantitative difference in protein contents measured by peak area (volume). Colour scheme ranges from purple (low absorbance) to red (high absorbance). The difference between absorbencies is shown in the middle as either a red or green band, representing the sample with greater absorbance at a specific peak.

The proteomic profiling platform ProteomeLab™ PF 2D offers 2-dimensional fractionation in which intact proteins are first separated by chromatofocusing proteins by pI and separated in the second dimension by their hydrophobic properties. The pH profiles from the chromatofocusing absorbencies were obtained from first dimension separation at 280 nm and thirty fractions were selected from each sample to submit for second dimension separation by hydrophobic chromatography. Second dimension absorbance profiles were compiled and displayed as a two-dimensional map using a feature of Mapping Tools software. The map displays pI fractions as lanes with the colour intensity of each band (absorbance at 214 nm) corresponding to protein bands located at their retention time of the second dimension separation (Figure 3A and 3B, Table 1).

**Table 1:** Summary of fractions selected for mass spectrometry analyses.

Fraction ID** (Fraction Coordinates)	Patient 1		Patient 2		Patient 3	
	
	GA Immunization					
	
	Before **(B)**	After **(A)**	Before **(B)**	After **(A)**	Before **(B)**	After **(A)**
F1 (pH: 5.90-5.60, RT:16.04–16.71)	1.49*	1.11	1.76	1.49	1.41	1.46
F2 (pH: 7.10-6.80, RT:14.03–14.70)	0.004	0.006	0.023	0.009	0.008	0.009
F3 (pH: 6.20-5.90, RT:14.70–15.37)	0.043	0.049	0.06	0.049	0.03	0.031
F4 (pH: 6.50-6.20, RT:16.04–16.71)	0.28	0.343	0.57	0.533	0.23	0.11
F5 (pH: 5.90-5.60, RT:16.71–17.38)	0.178	0.171	0.507	0.24	0.134	0.133
F6 (pH: 6.20-5.90, RT:15.37–16.04)	0.01	0.02	0.1	0.09	0.06	0.03
F7 (pH: 4.99-4.88, RT:14.70–15.37)	0.24	0.49	0.48	0.805	0.51	0.46
F8 (pH: 6.80-6.50, RT:15.37–16.04)	0.002	0.016	0.054	0.04	0.01	0.005
F9 (pH: 6.80-6.50, RT:16.04–16.71)	0.11	0.03	0.045	0.065	0.02	0.01
F10 (pH: 6.80-6.50, RT:14.03–14.70)	0.005	0.006	0.024	0.010	0.0086	0.009

Second dimension fractions representing selected pH ranges and retention times (RT). Corresponding peak heights are listed as absorbance units at 214 nm (*). Although there may be several peaks/fraction, values represent the peak having the greatest absorbance in the corresponding fraction. Fraction IDs are used in Table 2 (**).

Challenges associated with peak alignment are similar to those found during the alignment of spots in the analysis of 2-dimensional gel electrophoresis. Comparison of two separate UV/pI maps consisting of the entire pH gradient was afforded by a module (DeltaVue) of the Mapping Tools data processing software. A second module (MultiVue) enables the analysis of a selected pH lane from multiple sample runs. In both methods, proper peak alignment between samples provides a critical analytical function. Using the Paired Peak function of MultiVue in Mapping Tools, second dimension chromatograms were aligned by setting the paired peak value at +/- 0.5% to initially determine if peaks should be paired. After confirmation of automatic pairing, minor manual adjustments of retention times (RT) were made to accommodate alignment protocols. For example, raw data from one analyzed peak (fraction corresponding to pH 5.89-5.59 and RT 16.04–16.71) yielded a raw range of 8.4 seconds in RT throughout six samples. However, following alignment adjustments, the range was within 4.2 seconds, providing greater assurance when selecting peaks for quantitative and qualitative analysis.

**Figure 4:**
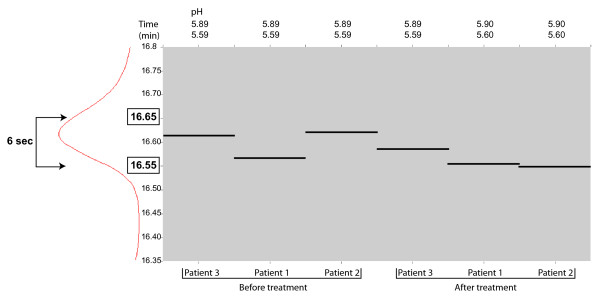
**RP-HPLC**. Differences in retention times (RT) (second dimension) displayed as bands for a specific peak detected and aligned in 6 samples using MultiVue software. The displayed peak corresponds to Patient 3 before treatment and has a RT of 16.62 min. Range of retention times was less than 6 sec.

Our data indicate that alignment and comparisons of the first dimension separation profiles were consistent. Alignment in second dimension, RP-HPLC, was also precise as differences in retention times between samples were in a range of less than 15 seconds. Figure 4 shows an example in which differences in retention times are less than 6 sec (0.1 min). Although automatic alignment is a standard software feature utilized in these studies for course alignment, manual alignment has proven necessary to refine peak analysis. Protein identification by LC-MS/MS presented in Table 2 afforded a level of confidence in our (manual) method of alignment. Nevertheless, alignment, whether automatic or manual, should be used with caution and peak identification must be validated.

**Table 2:** Summary of results from mass spectrometry analyses.

**Protein No**	**Protein**	**Fraction ID: Patient Sample**	**MW**	**NCBI Accession #**	**pI**
1	Hemopexin	F1: 1A,2A,3B,3A F8: 1B,1A,2B,2A,3B F6: 1B,1A,2B,2A,3B,3A F5: 1B,1A,2B,2A,3B,3A F7: 1B,1A,2B,3B	51676	11321561	6.55
2	Plasminogen	F1: 1A,2A,3B,3A F4: 1A,2A,3B,3A F6: 2^a^	90569	4505881	7.04
3	Anti-thrombin	F1: 2A,3B	13788	23978644	5.91
4	Histidine-rich glycoprotein precursor	F1: 1B,1A,3B F4: 1B,1A,2B,2A,3B,3A F9: 2B	59578	4504489	7.09
5	Complement Factor I	F5: 1A,2B,2°	65720	119392081	7.72
6	Complex of the Catalytic Domain of Human Plasmin and Streptokinase	F1: 1A,2A,3B F4: 1A,2B,2A,3B,3A F9: 2B	27286	5821850	8.27
7	Prealbumin	F1: 2A F5: 1B,2B,2A,3B	15919	219978	5.52
8	A Chain A, prealbumin	F1: 2A F8: 1B F5: 1B,2B,2A,3B	13760	230651	5.55
9	Beta-2 glycoprotein I apolipoprotein H	F1: 1A,3B F4: 1A,2A F6: 1A,2B,2A,3B	38312	28810	8.34
10	Gelsolin isoform a precursor	F1: 3B F9: 2B F6: 2B F5: 1B,3B,3A	85697	4504165	5.90
11	Kringle 2 Domain of Human Plasminogen	F4: 1A,2A,3B	9637	6573460	7.55
12	Complement factor H-related protein 1 precursor (FHR-1)	F6: 1A,2B,2A,3B F7: 2A	37661	543981	7.75
13	H factor (complement)-like 3	F6: 2A	30651	5031695	6.00
14	Retinol-binding protein 4, plasma precursor	F1: 2B F5: 2B	23010	55743122	5.76
15	Coagulation factor XII-Mie	F5: 2A	67735	24899162	8.03
16	Glutathione peroxidase 3 precursor	F5: 2A	25505	121672	8.20
17	Factor H	F7: 2A	139125	31965	6.28

Identification by LCMS/MS of proteins in plasma from three ALS patients before (B) and after (A) GA immunization. Only proteins with two or greater sequenced peptides are listed. Coordinates for Fraction IDs are listed in Table 1.

When comparing corresponding fractions from different samples, differences in retention times become critical for peak resolution since fraction collection intervals are determined prior to profile analysis. Therefore, a difference of several seconds may result in one peak being split into two fractions in one sample while collected entirely in one fraction for another. This is an inherent issue with LC based separation, whether stand-alone or in combination with other modes of separation. Thus, methods of separation and collection must be considered at the time when parameters are first set. One of several approaches to analyzing divided peaks involves determining the protein composition of each fraction. Another approach is to pool both adjacent fractions to increase the chances of protein identification from the corresponding split peak. However, pooling fractions may create more complexity in the sample and affect further analysis, such as increasing the number of proteins identified within the pool.

### Protein identification

High confidence protein identification is an essential step in most proteomic studies. The ProteomeLab™ PF 2D, unlike 2D DIGE, offers the added advantage that collected fractions are in liquid phase and can be utilized directly for any of various analytical procedures, such as mass spectrometer analysis, enzymatic digests, additional fractionation, Western blot, or a combination of analytical tests. Additionally, more material can be fractionated using 2D LC (up to 5 mg with the PF 2D) than with gel electrophoresis, thus significantly increasing the sensitivity of protein identification.

In the first step of this study, we investigated the correlation between detection of individual plasma fractions by UV absorbance and our ability to identify proteins with high confidence (two or more peptides). We digested proteins in a given plasma sample with trypsin and analyzed the digest for resulting peptides using nano-LC-MS/MS sequencing. Table 1 summarizes fractions with analogous pH, retention time, and similar peak height absorbance chromatograms. Selected fractions were digested and analyzed by nano-LC-MS/MS. Fractions with a peak height greater than 0.100 absorbance units at 214 nm provided enough material to identify proteins with high confidence using our instrumentation. Proteins in fractions with peak height below 0.050 were either identified with low confidence or remained unidentified. Proteins in fractions within the intermediate peak height range of 0.05 and 0.10 appeared to be the lower limit of identification by nano-LC-MS/MS. Table 2 displays only the proteins identified with high confidence from examined fractions. Using both automatic and manual alignment protocols, the same proteins were identified among several fractions across all six analyzed samples. These results indicated that using automatic alignment with minor manual adjustments provides enough confidence to pool corresponding sample fractions with low protein content and use them for high confidence protein identification without risk of mixing neighbouring peaks. The capacity to provide peak pooling enhances the utility of this proteomic platform.

### Fourth dimension fractionation

**Figure 5:**
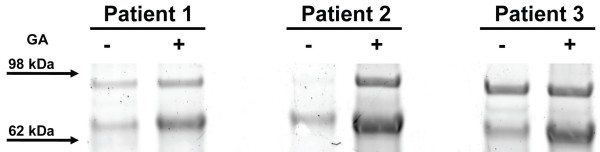
**1D Electrophoresis**. 1D electrophoresis (fourth dimension fractionation) showing differential expression of protein band above 62 kDa m.w. marker. Hemopexin was identified as the most prominent protein in this band by LCMS/MS sequencing. Equal amount of protein (based on absorbance at 214 nm) from each sample was loaded per lane. Gel was stained with SyproRuby.

One of the advantages of the PF 2D profiling platform is a possibility of fractionation in the fourth dimension. In our experimental design, IgY immunodepletion (partitioning) served as first dimension partitioning, isoelectric focusing provided second dimension analysis, and RP-HPLC yielded the third dimension. Several fractions after 3 dimensional analyses were selected for a fourth dimension, 1-dimensional electrophoresis (1DE), to evaluate whether the fraction isolated as a single peak consisted of only one protein. Frequently, fractions (covering a retention time of 0.67 min) with high peak height contain several proteins whose quantities are opposite to each other, thus masking differential expression. We selected one matching fraction from all six samples (pH 5.9 to 5.6, retention time 16.04 to 16.71 min.) with peak height ranging between 1.11 and 1.76 (Table 1). Analysis of these six matching fractions based on levels of absorbance after 3-dimensional fractionation did not demonstrate statistical differences between fractions, thus indicating that the total amount of protein in each fraction was the same. We expected and further confirmed by 1 DE that these fractions consisted of multiple, non-separated, proteins. Therefore, in a subsequent step, we analyzed by 1 DE equal amounts of each fraction containing equal amounts of protein based on absorbance at 214 nm. As expected, this analysis showed multiple bands in each fraction (Figure 5). A characteristic pattern showed increased intensity of a protein band with a molecular mass above 62 kDa in samples from immunized patients. It is possible that using narrower pH fractionation, e.g. 0.1 units instead of 0.3 units used in this study, would help to further separate proteins. This band was identified as hemopexin based on nano-LC-MS/MS analysis of peptides derived from tryptic digests and demonstrates that levels of this protein are increased in patients immunized with GA. The most extensively studied function of hemopexin is its binding heme, having the highest affinity of any known protein. Also, as a heme scavenger, hemopexin protects organisms from the oxidative damage that can be caused by free heme. Interestingly, histidine-rich glycoprotein precursor, whose function is not understood and which has been found in this study, is also a heme binding protein. Both proteins are made by liver and secreted to plasma.

Molecular mechanism(s) underlying ALS remain enigmatic and no curative or ameliorative therapy exists for this disorder that leads to degeneration of upper and lower motor neurons and ultimate death. Because ALS appears to be multifactoral, involving interactions among microglia, astrocytes, neurons, and muscles, one can anticipate that efficacious therapy would require a number of therapeutic approaches involving adjunct modalities that target several pathways associated with microglia activation and motor neuron degeneration. One immunomodulatory strategy using a synthetic polymeric immunogen, GA, which cross-reacts with CNS protein epitopes and is currently clinically utilized in the treatment of relapsing/remitting multiple sclerosis, targets several of these pathways [21]. In a recent phase II clinical trial, GA treatment of ALS patients proved to be safe and well tolerated [22]. Whereas significant diminution of lymphocyte proliferation was observed, subsequent studies showed increased concentration of anti-GA antibodies in plasma from treated patients [23]. To fully evaluate plasma differences in immunized patients compared to pre-immunized ALS patients and probe putative treatment targets for ALS, we employed a strategy of protein fractionation that allowed the separation of plasma proteins into several hundred fractions that are amenable to downstream evaluation.

## Discussion

ProteomeLab™ PF 2D is a new technology platform in proteomics [17-19,24-26] and literature reports using this platform have continued to emerge, totaling more than 40 to date. Our first approach discovered fewer differences than originally expected. However, the capacity for fourth dimensional separation and additional analysis provided an opportunity to further investigate selected fractions that otherwise would not have been available using the typical "bottom-up", non-recoverable tandem methods of separation and analysis used in conventional proteomics. This was possible because intact proteins were initially separated, analyzed and recovered in liquid phase. We were also able to identify isogenous fractions to pool when individual fractions contained insufficient amounts of protein for identification, a feature made possible by the ability to reproduce inter-sample fractionation patterns and precisely align peaks among replicate samples. Another strength of this platform is the flexibility to choose the number of fractions acquired per sample. We selected 30 first dimension fractions for second dimension separation, resulting in fractions covering a wide pH range and producing over 700 fractions per sample. However, the number of analyzed fractions and method parameters are within the investigator's prerogative. First, the collection of fractions based on the pH gradient can be adjusted to increase or decrease fractions collected; this is especially beneficial when the proteins of interest occur in a narrow pH range. Second, larger pH intervals for fraction collection may result in a higher number of proteins contained within each fraction, diminishing the separation of proteins and the potential for biomarker discovery. Narrowing pH intervals increases resolution, but will also increase the number of fractions to be analyzed in the second dimension, taking more time and further decreasing throughput. Similarly, the number of first dimension fractions to be run in the second dimension can be selected. Also, slower gradient elution from RP-HPLC columns can be utilized but will result in longer second dimension runs and requires some initial experimentation to optimize the desired analysis. Adjusting pH parameters and acetonitrile gradients may be useful, but needs to be applied with caution to assure the overall benefit from profiling.

We have yet to analyze fractions in great depth for PTM differences, although such modifications can be indicative of changes resulting from immunization. In particular, shifts in protein isoelectric point usually indicate changes resulting from PTMs [27]. Therefore, it is possible that the identification of similar proteins from a variety of pH and RT fractions results from PTMs and/or protein fragmentation, as was seen in our study. Other approaches of sample fractionation prior to profiling can be included. For example, use of "Equalizer^® ^beads" [28-30] might be an advantageous alternative or addition to broadly used immunodepletion of the most abundant proteins. Using lectin columns for isolation of subsets of post-translationally glycosylated proteins offers another approach to address challenges of profiling and discovery of biomarkers in a very complex mixture of proteins, especially when changes are subtle [31,32]. Still, given the sample recovery capacity and sample concentration, those studies are within the realm of this platform.

## Conclusion

In summary, we found the ProteomeLab™ PF 2D to be a useful automated proteomic profiling platform. There are several strengths of this approach. One advantage over the second dimension of 2-dimensional electrophoresis (2-DE) is the separation of proteins, which might be very similar in size but quite different in their biochemical characteristics due to post-translational modification. Also, this platform can be used for profiling basic and hydrophobic proteins that are hard to analyze by 2-DE [33,34]. Another advantage is that fractionated proteins are maintained in a liquid phase, making them available for various assays without loss of material (e.g., extraction from polyacrylamide gel) and/or allowing fractionation by other means. Moreover, due to the automated nature of this platform, the option of using only chromatofocusing for separation affords an attractive advantage over conventional gel-based separation by isoelectric focusing. Weaknesses of the ProteomeLab™ platform include low throughput, allowing 2–3 samples per week per instrument, and relatively large amount of sample required for analysis. Although multiple instruments can be run in parallel to increase throughput, this is very expensive. Large amount of sample is not an issue when serum/plasma samples are analyzed. However, other clinical material such as tissue biopsies might not be available in sufficient amounts. Also, the masking of differential protein expression among samples by opposite quantities of protein in a given fraction may present a problem when looking for potential biomarkers, as seen by the differential expression of hemopexin in our study. We have presented preliminary data regarding the effects of GA treatment for ALS and have found the ProteomeLab™ PF 2D to be a promising platform for protein profiling and a means for biomarker discovery.

## Methods

### Samples

Peripheral blood samples from ALS patients used in this study were obtained from Columbia University, New York. ALS patients were treated daily with 20 mg of GA over a six month period [22]. Samples were collected in acid citrate dextrose tubes and after centrifugation at 800 × *g *for 10 min, the plasma was harvested, distributed into 1 mL aliquots, and stored at -80°C. Plasma from 3 ALS patients (collected prior to treatment and 2, 4, and 5 months after initiation of GA treatment) was used in this investigation.

### Sample delipidation

Plasma samples were centrifuged at 18000 × *g*, 15 min at 4°C. The middle layer was collected and diluted 1:2.5 (0.25 mL plasma + 0.375 mL) in dilution buffer (10 mM Tris-HCl, pH 7.4, 0.15 M NaCl). Next, particles and aggregates were removed from samples by filtration through a 0.45 μm spin filter at 9200 × *g *for 1 min.

### Immunodepletion (partitioning)

To remove 12 highly abundant proteins, we utilized the ProteomeLab™ IgY-12 High Capacity Proteome Partitioning Kit (Beckman Coulter, Fullerton, CA) according to manufacturer's recommendations. The kit included a LC10 affinity column (12.7 × 79.0 mm) with a capacity of 0.25 mL human plasma per cycle and optimized buffers for sample preparation, loading, washing, and eluting. The LC10 column contains affinity-purified chicken IgY antibodies directed against serum albumin, fibrinogen, IgG, transferin, IgA, IgM, apoA-I, apoA-II, haptoglobin, α1-antitrypsin, α2-macroglobulin, and α1-acid glycoprotein, which are covalently conjugated to polymeric microbeads. After the enriched flow through fractions containing low to medium abundant proteins were collected, the bound and highly abundant proteins were eluted with stripping buffer (0.1 M Glycine-HCl, pH 2.5). The column was then neutralized with 0.1 M Tris-HCl, pH 8.0 buffer. Finally, the column was re-equilibrated with dilution buffer at a flow rate of 2 mL/min. Collected bound fractions were neutralized with 0.1 M Tris-HCl. Flow through and eluted fractions were stored at -80°C until further analysis.

### Sample preparation for PF 2D first dimension (isoelectric focusing)

Collected flow through fractions were thawed at room temperature and concentrated down to 0.50 mL using an Amicon Ultra-15 centrifugal filter (Millipore, Billerica, MA) previously washed with 3 mL of ProteomeLab™ Start Buffer (Beckman Coulter, pH 8.5). Next, 2 mL of plasma denaturing buffer (7.5 M urea, 2.5 M thiourea, 12.5% glycerol, 62.5 mM Tris-HCl, 2.5% (w/v) n-octylglucoside, 1.25 mM EDTA) was added to the concentrator and left at room temperature for 30 min while shaking. Samples were removed and centrifuged using two 1.5 mL screw-cap microcentrifuge tubes at 15,000 × *g *for 1 hr at 18°C. PD-10 Desalting Columns (GE Healthcare) were prepared by equilibration with 25 mL of Start Buffer. Sample was removed from the plasma denaturing buffer and placed into Start Buffer by placing sample load (2.5 mL) onto PD-10 column, discarding effluent, and collecting the desalted sample with 3.5 mL of Start Buffer. Resulting sample was filtered through a 0.45 μm spin filter previously washed with the Start Buffer. Protein concentration was determined by the Micro BCA Assay Kit (Pierce Biotechnology, Rockford, IL).

### Profiling

Protein profiling using ProteomeLab™ PF 2D system consists of two steps: first dimension fractionation is chromatofocusing and second dimension is reverse phase HPLC fractionation. The 32 Karat™ Software (Beckman Coulter) was used for data processing and calculation of peak areas and heights.

### First dimension fractionation

The first dimension was performed at room temperature with a flow rate of 0.2 mL/min using the HPCF column Start Buffer (pH 8.5), Eluent Buffer (pH 4.0), High Ionic Strength Wash + (1 M NaCl in 30% Isopropanol), and water. It was performed at room temperature with a flow rate of 0.2 mL/min. After equilibration with the Start Buffer for 130 min, the samples (1–5 mg of protein) were injected onto the chromatofocusing column and proteins separated based on isoelectric point (pI). Thirty-five min after injection, Eluent Buffer was initiated to generate a pH gradient (8.5–4.0). Shortly after the gradient reached pH 4.0, the column was washed with HISS+ (135–175 min after injection) to remove hydrophobic proteins and proteins with a pI under 4.0. Finally, the column was washed with water for 45 minutes (175–220 min after injection). Proteins were detected by absorbance at 280 nm by a UV detector. Based on the pH gradient generated, fractions were collected with the fraction collector/injector module (FC/I) at 0.3 pH intervals during pH gradient elutriation (8.3–4.0), otherwise fractions were collected every 8.5 min.

### Second dimension fractionation

The second dimension separations were performed with an RP-HPLC column and two solvents, 0.1% trifluoroacetic acid (TFA) in HPLC water (Solvent A) and 0.08% TFA in acetonitrile (ACN) (Solvent B). Separation was executed at 50°C at a flow rate of 0.75 mL/min and protein containing fractions detected by UV absorbance at 214 nm. Equilibration was achieved with Solvent A for 10 minutes followed by Solvent B for 5 minutes prior to each injection. From selected first dimension fractions, 0.250 mL were injected, run for two minutes, and the column eluted with a linear gradient of 0–100% Solvent B for 30 min. (3.33% change in B solvent/min). Next, Solvent B was continued for four minutes, followed by re-equilibration with 100% Solvent A for eight minutes. Second dimension fractions were collected at 40 second intervals.

### In solution trypsin digestion

The amount of protein from each fraction used for digestion was determined based on peak UV absorbance at 214 nm. Samples were dried to 10 μL followed by addition of 50 mM ammonium bicarbonate and DTT. After 1 hr incubation in a 60°C water bath, trypsin (0.125 mg/mL) was added and samples incubated at 37°C for 14–16 hours. Trypsinized samples were sonicated using a Branson water bath sonicator for 5 seconds, a second volume of trypsin added, and samples incubated at 37°C for 8–10 hours. Formic acid was added to a final concentration of 0.1% to stop the reaction. Peptides were purified using ZipTip^® ^(Millipore Corporation, Billerica, MA) according to manufacturer's recommendation. Eluates were dried by vacuum centrifugation and resuspended in 12 μL of HPLC water with 0.1% formic acid prior to mass spectrometer analysis.

### Protein identification by nano-LC-MS/MS

Peptides were fractionated on a RP-C18 microcapillary column and sequenced using electrospray ionization-liquid chromatography-mass spectrometry system (ESI-LC-MS/MS) (ProteomeX system equipped with LCQDeca*XP*Plus mass spectrometer, ThermoElectron, Inc., San Jose, CA) in a nanospray configuration. The mass accuracy of the LCQDeca*XP*Plus is 500 ppm +/- 100 ppm. Database nr.fasta was retrieved from the NCBI FTP server  updated on April 11^th^, 2008. The spectra obtained through LC-MS/MS analysis were searched against the protein database narrowed to a subset of human proteins (keywords: Homo sapiens, man, human, primate) using SEQUEST algorithm (BioWorks 3.2 software from ThermoElectron, Inc.). We excluded keratins from our database search based on previous observations that these are contaminants resulting from sample processing. In TurboSEQUEST Search Parameters, threshold for Dta generation was 10000 and precursor mass tolerance for Dta generation was set at 1.4. For Dta Search, peptide tolerance was set at 1.5 and fragment ions tolerance at 0.00. Charge state was set on "Auto." At least two sequenced peptides were required from each protein for high confidence identification.

### SDS-PAGE

Selected second dimension protein fractions were resolved further by electrophoresis through a NuPAGE^® ^Novex 4–12% Bis-Tris Gel (Invitrogen, Carlsbad, CA, USA). Fractions were dried, resuspended in 20 μL of 1× sample buffer with reducing agent (Invitrogen), and heated at 95°C for 5 minutes. Electrophoresis was performed at a constant 100 V for 90 min and the gel was fixed in 40% methanol/7% acetic acid. After staining with SYPRO^® ^Ruby protein gel stain (Invitrogen), destaining was achieved with 10% methanol/7% acetic acid and the gel scanned using a Typhoon 9410 high performance laser scanning system (GE Healthcare/Amersham, Piscata, NJ). Gels were subsequently counterstained with Coomassie Brilliant Blue G-Colloidal (Sigma, St. Louis, MO) and bands excised for in gel tryptic digestion.

### In gel trypsin digestion

Gel pieces were distained for 1 hr at room temperature using 100 μL of 50% acetonitrile/50 mM NH_4_HCO_3_. Gel pieces were dried and incubated with trypsin in 10 mM NH_4_HCO_3 _(Promega, Madison, WI) overnight at 37°C. Peptides were extracted from trypsinized gels by washing gel pieces for 2 hours with 0.1% TFA and 60% ACN and were purified using ZipTip (Millipore Corporation). ZipTip eluates were dried and resuspended in 12 μL of HPLC water with 0.1% formic acid prior to mass spectrometer analysis.

## Competing interests

The authors declare that they have no competing interests.

## Authors' contributions

JDS has made substantial contributions to data acquisition and analysis. Has been involved in drafting the manuscript. WR has made substantial contributions to data acquisition and analysis. RS has made substantial contributions to conception and design. Has been involved in revising manuscript critically for important intellectual content. RLM has been involved in collecting samples for this study. Has been involved in drafting the manuscript and revising it critically for important intellectual content. HEG has made substantial contributions to conception and design. Has given final approval of the version to be published. PC has made substantial contributions to conception and design, data analysis and interpretation. Has been involved in drafting the manuscript and revising it critically for important intellectual content. All authors have read and approved the final manuscript.
